# Disproportionate Burden of COVID-19 Infection Among Hispanic Patients During the First COVID-19 Surge in South Texas

**DOI:** 10.1089/heq.2021.0185

**Published:** 2022-07-22

**Authors:** Meredith G. Hosek, Aditi Sharma, Mary Nunn, Sydney T. Tran, Madeleine O. Bousquet, Zachary T. Allen, Farah A.L. Allawi, Robert Geller, Andrew McCracken, Carmen G. Sanchez, Anna G. Taranova, Roberto Villarreal, Martin Goros, Jonathan Gelfond, Barbara S. Taylor

**Affiliations:** ^1^ Long School of Medicine, University of Texas Health San Antonio, San Antonio, Texas, USA.; ^2^ Department of Internal Medicine, University of the Incarnate Word School of Osteopathic Medicine, San Antonio, Texas, USA.; ^3^ Department of Population Health Sciences, University of Texas Health San Antonio, San Antonio, Texas, USA.; ^4^ Department of Research & Information Management, University Health, San Antonio, Texas, USA.; ^5^ Department of Clinical & Business Analytics, University Health, San Antonio, Texas, USA.; ^6^ Department of Public Health, Innovation, and Equity, University Health, San Antonio, Texas, USA.; ^7^ Department of Epidemiology and Biostatistics, University of Texas Health San Antonio, San Antonio, Texas, USA.; ^8^ Department of Medicine, University of Texas Health San Antonio, San Antonio, Texas, USA.

**Keywords:** COVID-19, COVID-19 transmission, health disparity, ethnic disparity, Hispanic health, South Texas

## Abstract

**Introduction:**

The morbidity and mortality of the COVID-19 pandemic have disproportionately burdened Hispanic populations in the United States. While health equity research is typically conducted in populations where Hispanics are the minority, this project analyzes COVID-19 racioethnic transmission trends over the first 6 months of the pandemic within a large majority-minority city in South Texas.

**Methods:**

Patients diagnosed with COVID-19 across inpatient, emergency department, and outpatient settings of a large county health system were included in a clinical registry. For 4644 COVID-19-positive patients between March 16 and August 31, 2020, demographic and clinical data were abstracted from the registry. Race/ethnicity trends over time were compared for patients with and without COVID-19 diagnoses. Logistic regressions identified predictors of inpatient diagnosis by age, race/ethnicity, and testing delay.

**Results:**

The proportion of patients with COVID-19 identifying as Hispanic increased rapidly during the pandemic's first months: from 55.6% in March to 85.7% in June. A significantly greater proportion of patients identified as Hispanic within the COVID-19 cohort compared to other diagnoses cohort. Testing delay was 11.6% longer for Hispanic patients, with each day of testing delay associated with 7% increased odds of inpatient COVID-19 diagnosis.

**Conclusion:**

These findings highlight the disproportionate impact of COVID-19 on Hispanic populations even within a majority-minority community. In the United States, Hispanic persons are more likely to work frontline jobs, live in multigenerational homes in poverty, and be uninsured. The burden of COVID-19 cases within Bexar County's largest hospital system reflects this systemic inequity. Identifying racioethnic health disparities supports efforts toward mitigating structural factors that predispose minority groups to illness and death.

## Introduction

Since the onset of the COVID-19 pandemic, communities of color have borne a disproportionate burden of risk, morbidity, and mortality in the United States.^1^ The current crisis both highlights and exacerbates the long-standing health disparities by race and ethnicity in the United States.^2^ During early summer 2020, it is estimated that hospitalization rates among racial and ethnic minorities were 4 to 6 times that of White non-Hispanic individuals.^3^ Understanding the dynamics of inpatient COVID-19 diagnoses within majority-minority communities can help elucidate these disparities and offer insights to help reduce inequity in this pandemic and beyond.

Relaxation of stay-at-home measures occurred earlier in the pandemic in Texas than in most other states. Reopening of nonessential retail businesses began in May 2020 and continued through mid-June, at which point most businesses were open at 75–100% capacity.^4^ Although seemingly ethnically neutral, these reopening policies disproportionately affected communities of color. Notably, essential workers in Texas are two times more likely to be African American and 1.3 times more likely to be Hispanic compared with non-Hispanic Whites.^5^

In addition, before the pandemic, only 16.2% of Hispanic workers in the United States had jobs that allowed them to work from home.^6^ Multiple socioeconomic factors add to the disproportionate risk of infection in Hispanic communities. Hispanic persons are more likely to live within multigenerational homes, making social distancing difficult and transmission more likely.^6^ Hispanic persons also have the highest rate of under-insurance in the country, higher burden of chronic conditions, and language barriers that can impede access to health care.^6^ This combination of exposures and risk, combined with structural factors like discrimination, socioeconomic gaps, and crowded housing, led to a perfect storm for Hispanic communities during the first surge of COVID-19 in Texas.

The multiple structural factors leading to increased risk for Hispanic communities are reflected in the disproportionate burden of COVID-19 infection, hospitalization, and death in the Hispanic community.^1^ These disparities were reflected in national data from March to May 2020, when COVID-19 test positivity rates rose among Hispanic and Black Non-Hispanic persons, while falling among White non-Hispanic persons.^7^

By June 2020, 33% of COVID-19 cases in the United States were among Hispanic individuals, even though they comprised 18.5% of the population.^8^ This early disparity persisted with ∼27.6% of cases to date occurring in Hispanic persons.^9^ In June 2020, 96.2% of hotspot counties for COVID-19 transmission had racioethnic disparities in COVID-19 incidence, with Hispanic/Latino persons comprising the largest racioethnic group by population size within hotspot counties.^10,11^ Data from the National Center for Health Statistics in the United States through November 2021 reveal that Hispanic persons have been 2.8 times more likely to be hospitalized and 2.3 times more likely to die from COVID-19 infection than non-Hispanic Whites.^12^

Most research demonstrating the disproportionate impact of COVID-19 on Hispanic communities has been conducted within communities where Hispanics are a minority racioethnic group. Bexar County, which contains San Antonio, is the largest population center of South Texas (2.01 million people) and a minority-majority county where 60.7% of the population is Hispanic, significantly higher than Texas (39.7%) and the nation (18.5%).^13^ This study utilizes a prospective, longitudinal clinical registry of all patients diagnosed with COVID-19 within the public safety net hospital system serving Bexar county to examine disparities in COVID-19 outpatient, emergency department (ED), and inpatient diagnoses by ethnicity. In addition, this analysis focuses on racioethnic trends of COVID-19 diagnoses within a public health care system during the first 6 months of the pandemic, a time frame heavily influenced by the lifting of stay-at-home orders and lockdown restrictions.

## Methods

### Context of University Health and the COVID-19 ID Virtual Outpatient Clinic

University Health is the third-largest public health system in Texas, serving nearly 300,000 people annually, regardless of ability to pay.^14^ As a political subdivision of the State of Texas and a Level I Trauma Center, University Health provides services to Public Health District Region 8, a 28-county region with the highest rates of uncompensated care of Texas' 11 public health districts.^15^ The region covers 37,800 square miles, stretches along the Mexico border, and serves over 3.03 million people or 10.4% of the state.^16^ Since 2020, University Health became a COVID-19 testing and vaccination hub for South Texas, providing over 550,000 vaccine doses and 252,000 COVID-19 tests.

The COVID-19 Infectious Disease Virtual Outpatient Clinic (CIVOC) was developed in March 2020 to provide virtual follow-up care for people with COVID-19 infection. The CIVOC dataset and clinic operations were administered through Research Electronic Data Capture (REDCap; Vanderbilt University, Nashville, TN), with templates adapted from a similar COVID-19 virtual outpatient clinic developed at New York Presbyterian Hospital, Columbia University Medical Center. Patients testing positive for SARS-CoV-2 in the Bexar county public health safety net system across outpatient, ED, and inpatient settings had their basic demographic information (i.e., age, race/ethnicity) and date of positive test extracted from the electronic health record (EHR) into a REDCap database.

### Context of the COVID-19 pandemic

In October 2020, MetroHealth, the public health agency responsible for Bexar County, published a report of COVID-19 statistics for the first 6 months of the pandemic. The 7-day case average ranged from ∼200 to 1200 in late June 2020. Test positivity rates ranged from 5% in May to 24.2% in early July. Hispanics comprised 75.8% of cases, while Non-Hispanic Whites, Non-Hispanic Blacks, and Asians made up 17.4%, 5.1%, and 1.5%, respectively. Hispanics' disproportionate representation was consistent within each 10-year age group category.^17^ By April 2020, multiple free testing sites not requiring provider referral were available throughout San Antonio sponsored by MetroHealth and University Health, expanding weekly testing capacity from under 3000 to a peak of 24,437 in July.

### Study population and inclusion criteria

Patients included in the COVID-19 cohort tested positive in settings affiliated with University Hospital between March 16, 2020, and August 31, 2020. Patients with missing ethnicity data in the EHR (Blank, Unknown, and Decline to Answer) were excluded unless race was listed as Hispanic, in which case, they were included as Hispanic (433 of 4644 patients excluded). The study team verified race, ethnicity, and date of birth for all patients within the COVID-19 cohort by searching for their medical record number in the EHR to confirm the integrity of the data transfer into the CIVOC registry. Date of symptom onset was sometimes collected as part of the virtual clinical encounter.

Before June 3, 2020, persons admitted to University Hospital were only tested for COVID-19 if clinically indicated. After June 3, 2020, all existing inpatients and inpatient admissions were tested for COVID-19 regardless of symptoms.

Over the same observation period and at the same inpatient and outpatient clinics, deidentified patient data from all visits without a COVID-19 diagnosis were gathered for comparison with the COVID-19 cohort, including race, ethnicity, age, and date and location of the clinical encounter.

### Covariate and outcome definitions

Predictive variables extracted from the EHR included age at the time of COVID diagnosis and a combined patient-identified race/ethnicity variable, categorized as follows: Hispanic, White non-Hispanic, Black non-Hispanic, or Other. COVID-19-related predictors extracted were COVID-19 positive test date and date of symptom onset.

The primary outcome was the proportion Hispanic patients in the COVID-19 cohort, by month, compared with the non-COVID-19 cohort. As a secondary outcome, researchers evaluated non-Hispanic Black representation across the two cohorts. Additional secondary outcomes included testing delay, calculated as the number of days between the date of symptom onset and positive COVID-19 test, and location of COVID-19 diagnosis, categorized as outpatient/ED or inpatient.

### Statistical analysis

The racioethnic breakdown of patients with a COVID-19 diagnosis was compared to the breakdown of patients evaluated in the same settings without a COVID-19 diagnosis over the same time period using chi-squared tests and Fisher's exact test to assess for disparities by month. A chi-squared test was also conducted to compare percent Hispanic patients between the COVID-19 diagnosis cohort with the non-COVID-19 diagnosis cohort. A second chi-squared analysis was then conducted to compare percent non-Hispanic Black patients between the COVID-19 diagnosis cohort and the non-COVID-19 diagnosis cohort.

*t*-Test on the geometric means was used to assess testing delay of Hispanic patients of any race with non-Hispanic patients of any race as referent. A secondary analysis with one-way analysis of variance of testing delay with a four-level race/ethnicity variable (non-Hispanic White, non-Hispanic Black, Hispanic, and other) was then conducted with non-Hispanic White patients as referent, comparing testing delay among non-Hispanic White patients to testing delay among Hispanic patients, non-Hispanic Black patients, and Other race patients. Logistic regression was used to identify variables that predicted inpatient COVID-19 diagnosis versus outpatient/ED COVID-19 diagnosis. Variables assessed by logistic regression included age, race/ethnicity, and testing delay. Within this logistic regression, we also tested whether race/ethnicity might have moderated the effect of age by testing the statistical interaction between these variables.

### Ethical considerations

This research was reviewed by the Institutional Review Board of the University of Texas Health Science Center San Antonio (Protocol HSC20200772E) and was determined to be exempt on November 24, 2020.

## Results

### Summary of patient demographics

The majority of patients in the longitudinal cohort identified as Hispanic (83.0%) and female (54.5%) with a mean age of 41.6 years (standard deviation=17.8). Most patients with COVID-19 during this time frame were diagnosed during June and July 2020, consistent with the larger surge of COVID-19 infections in Bexar County.^18^ Demographics, including race/ethnicity, sex, age, and month of diagnosis, are presented by location of initial diagnosis (inpatient vs. ED/outpatient) in Table 1.

**Table 1.** Summary of Patient Demographics by Location of Initial Positive SARS-CoV-2 Test

Characteristic	Total (***n***=4644)	Inpatient diagnosis (***n***=815)	Outpatient/ED diagnosis (***n***=3829)
Age (mean, SD)	41.6 (17.8)	50.9 (18.5)	39.6 (17.0)
Race/ethnicity (*n*, %)			
Hispanic	3497 (83.0)	620 (79.2)	2877 (83.9)
Non-Hispanic White	451 (10.7)	107 (13.7)	344 (10.0)
Non-Hispanic Black	163 (3.9)	45 (5.7)	118 (3.4)
Asian	58 (1.4)	6 (0.8)	52 (1.5)
Other	42 (1.0)	5 (0.6)	37 (1.1)
Sex (*n*, %)			
Female	2530 (54.5)	382 (46.9)	2148 (56.1)
Male	2060 (44.4)	424 (52.0)	1636 (42.7)
Other	2 (0.1)	2 (0.2)	0 (0.0)
Month of diagnosis (*n*, %)			
March 2020	37 (0.8)	17 (2.1)	20 (0.5)
April 2020	131 (2.8)	39 (4.8)	92 (2.4)
May 2020	114 (2.5)	40 (4.9)	74 (1.9)
June 2020	1799 (38.7)	265 (32.5)	1534 (40.1)
July 2020	1925 (41.5)	317 (38.9)	1608 (42.0)
August 2020	638 (13.7)	137 (16.8)	501 (13.1)

ED, emergency department; SD, standard deviation.

### Proportion of Hispanic patients with and without COVID-19

The proportion of patients who tested positive for COVID-19 within the cohort identifying as Hispanic increased rapidly over the few months of the pandemic (Table 2). In March 2020, Hispanic patients accounted for 52.9% of inpatient COVID-19 diagnoses and 58.0% of outpatient diagnoses. By June 2020, however, patients identifying as Hispanic accounted for 86.1% of inpatient COVID-19 diagnoses and 85.7% of outpatient diagnoses.

**Table 2.** Number and Proportion of Patients with COVID-19 Diagnosis Versus Patients without COVID-19 Diagnosis by Race and Ethnicity Over Time, Including Inpatient, Outpatient, and Emergency Department Diagnoses

	COVID-19 diagnosis (***n***, %)	No COVID-19 diagnosis (***n***, %)	***p***
March 2020			0.058^*^
Hispanic	20 (55.6)	30,725 (70.3)	
Non-Hispanic White	9 (25.0)	7303 (16.7)	
Non-Hispanic Black	6 (16.7)	3168 (7.2)	
Other	1 (2.8)	2504 (5.7)	
April 2020			0.388^*^
Hispanic	95 (75.4)	59,696 (71.0)	
Non-Hispanic White	22 (17.5)	13,930 (16.6)	
Non-Hispanic Black	6 (4.8)	6022 (7.2)	
Other	3 (2.4)	4384 (5.2)	
May 2020			0.015^†^
Hispanic	76 (71.0)	66,854 (69.3)	
Non-Hispanic White	11 (10.3)	16,373 (17.0)	
Non-Hispanic Black	15 (14.0)	6908 (7.2)	
Other	5 (4.7)	6381 (6.6)	
June 2020			<0.001^†^
Hispanic	1456 (85.7)	75,075 (69.4)	
Non-Hispanic White	156 (9.2)	18,785 (17.5)	
Non-Hispanic Black	48 (2.8)	7565 (7.0)	
Other	38 (2.2)	6679 (6.2)	
July 2020			<0.001^†^
Hispanic	1372 (83.5)	64,798 (71.7)	
Non-Hispanic White	173 (10.5)	15,202 (16.8)	
Non-Hispanic Black	60 (3.6)	6240 (6.9)	
Other	39 (2.4)	4086 (4.5)	
August 2020			
Hispanic	478 (79.7)	59,631 (71.1)	<0.001^†^
Non-Hispanic White	80 (13.3)	14,697 (17.5)	
Non-Hispanic Black	28 (4.7)	5912 (7.1)	
Other	14 (2.3)	3590 (4.3)	
March–August 2020			
Hispanic	3497 (83.0)	356,779 (70.4)	<0.001^†^
Non-Hispanic White	451 (10.7)	86,290 (17.0)	
Non-Hispanic Black	163 (3.9)	35,815 (7.1)	
Other	100 (2.4)	27,624 (5.5)	

^*^ Fisher's exact test.

† Chi-squared test.

Table 2 compares the racioethnic breakdown of patients with and without COVID-19 during the observation period. Overall, there was a statistically significant difference in the race/ethnicity distribution of people diagnosed with COVID-19 and people with all other diagnoses in May (*p*=0.015), June (*p*<0.001), July (*p*<0.001), and August 2020 (*p*<0.001).

**FIG. 1.**
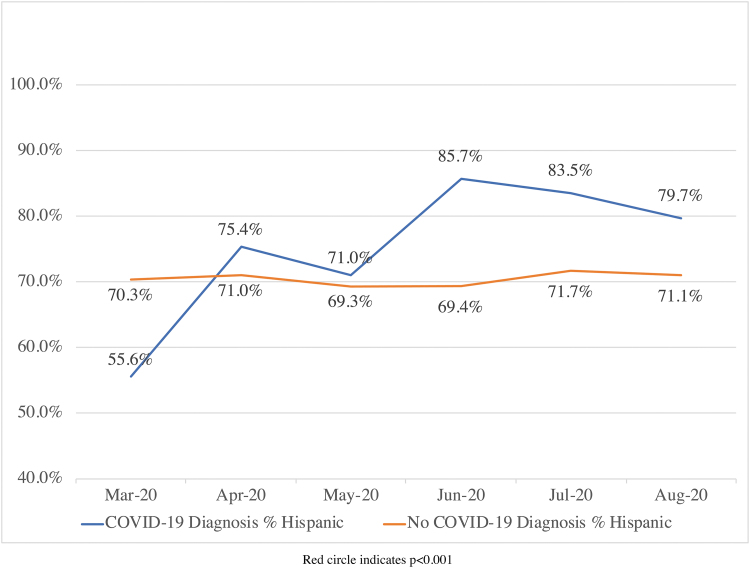
Proportion of patients presenting to care in the county health system, including outpatient/emergency department and inpatient settings, with COVID-19 diagnoses versus all other diagnoses, who identify as Hispanic.

Our primary analysis, comparing Hispanic representation between the two cohorts, demonstrated statistically significant differences only in the months of June, July, and August (*p*<0.001; Fig. 1). Most strikingly, in June 2020, while Hispanic patients represented 85.7% of all COVID-19 patients in the longitudinal cohort, they only comprised 69.4% of patients seen in the county health system for all non-COVID diagnoses (*p*<0.001). In March 2020, Hispanic persons comprised 55.6% of COVID-19 diagnoses and 70.3% of other diagnoses, but this was not statistically significant due to the small number of recorded COVID-19 cases in March 2020 (*p*=0.0.8).

There was no statistically significant difference between the proportion of Hispanic patients with and without COVID-19 in the months of April (*p*=0.328) or May (*p*=0.772). Overall, however, Hispanic patients represented 83.0% of COVID-19 diagnoses compared with 70.5% of all other diagnoses during the first 6 months of the pandemic, as captured in our health care network (*p*<0.001).

### Proportion non-Hispanic Black patients with and without COVID-19

When comparing the proportion of non-Hispanic Black patients in the COVID-19 cohort versus non-COVID-19 cohort, there was a greater proportion of non-Hispanic Black patients in the non-COVID-19 cohort in the months of May (*p*=0.01), June (*p*<0.001), July (*p*<0.001), and August 2020 (*p*<0.023). There was no significant difference between the two cohorts in March or April 2020. Overall, from March to August 2020, there was a greater proportion of non-Hispanic Blacks in the non-COVID-19 cohort than the COVID-19 cohort (7.1% vs. 3.9%, *p*<0.001).

### Testing delays by race/ethnicity

For patients with date of symptom onset available (*n*=1886, 40.6% of cohort), the number of days between date of symptom onset and initial positive COVID-19 test was 11.6% longer (95% confidence interval 0.3–24.2%, *p*=0.04) for persons identifying as Hispanic (*n*=1570, geometric mean=3.9 days) than for persons of any race identifying as non-Hispanic (*n*=316, geometric mean=3.5 days).

For the secondary analysis of testing delay with a four-level race/ethnicity variable (non-Hispanic White, non-Hispanic Black, Hispanic, and other), there was no significant difference in testing delay between non-Hispanic White patients (*n*=198, geometric mean=3.6 days) and Hispanic patients (*n*=1570, geometric mean=3.9 days), non-Hispanic Black patients (*n*=67, geometric mean=3.4 days), or patients of other races (*n*=51, geometric mean=3.5 days).

### Predictive variables for inpatient diagnosis

For every additional year of age, odds of inpatient diagnosis increased by 4% (*p*<0.001). For each day of testing delay, odds of inpatient diagnosis increased by 7% (*p*<0.001). Race and ethnicity, however, were not significantly associated with increased odds of inpatient diagnosis (Table 3). There was also no significant interaction between a patient's age and race/ethnicity.

**Table 3.** Logistic Regression of Years of Age, Minority Race or Ethnicity, and Days of Testing Delay in Predicting Inpatient Diagnosis Over Outpatient Diagnosis

Characteristic	OR	95% CI	***p***
Age	1.04	1.04–1.05	<0.001
Race/ethnicity			
Non-Hispanic White	—	—	
Non-Hispanic Black	1.29	0.63–2.54	0.5
Hispanic	0.85	0.58–1.25	0.4
Other	0.55	0.21–1.29	0.2
Days of testing delay^1^	1.07	1.05–1.10	<0.001

CI, confidence interval; OR, odds ratio.

## Discussion

This large, longitudinal study of patients within a county health system in a majority-minority city in Texas demonstrates that Hispanic representation in COVID-19 diagnoses increased during the first COVID-19 surge as public health restrictions eased.^4^ Hispanic patients with COVID-19 experienced a longer delay between symptom onset and testing, 11.6% longer than non-Hispanic patients. When testing delay of Hispanic patients was compared with non-Hispanic Whites in a four-variable race/ethnicity analysis, there was no significant difference. This may be due to a lack of power in the four-way analysis since the direction of association remained. Although we also found that testing delay was associated with an increased likelihood of inpatient diagnosis, we did not find an association between Hispanic ethnicity and inpatient diagnosis. Our investigation adds to the growing body of evidence that Hispanic communities in the United States have been disproportionately affected by the COVID-19 pandemic.

A strength of this analysis is that the COVID-19 cohort was compared with overall care-seeking behavior within the county health system, demonstrating the disproportionate representation of Hispanics among COVID-19 cases. Within a county that is 60.7% Hispanic, during the first 6 months of the pandemic, 83.0% COVID-19 cohort identified as Hispanic compared with 70.4% of patients who sought care for other causes in the same locations over the same time.^13^

This disproportionate burden of COVID-19 on the Hispanic community of South Texas in summer 2020 should be understood within the broader context of socioeconomic disparity, a context in which Hispanic persons are more likely to work front-line jobs, live in poverty, and dwell in multigenerational homes, all which compound risk of infection.^6^ Texas' essential workforce is disproportionately staffed by African American and Hispanic persons.^5^ In fact, 29% of Hispanic female workers and 13% of Hispanic male workers have jobs deemed essential.^5^ Understandably, as Texas eased restrictions in restaurants, entertainment, and retail, Hispanic persons were more likely to be exposed to the virus than White non-Hispanic persons in South Texas.

The COVID-19 testing delay found among Hispanic populations in this study can also be conceptualized within the greater framework of health care access disparities. In the United States, Hispanic populations are more likely to be uninsured than non-Hispanic Whites (20.0% vs. 7.8%).^19^ In Texas, the rates are even higher, with 30.8% of Hispanics lacking insurance compared to 13.0% of non-Hispanic Whites.^19^ In Bexar County, 43% of households speak Spanish, posing additional language barriers when accessing predominantly English-speaking health care systems.^20^ In addition, San Antonio has a large population of undocumented immigrants who may delay health care out of fear.^21^ Finally, in the historical context of health care injustices toward minorities and the prevalence of provider implicit bias against minorities, there is widespread mistrust of U.S. health care systems within the Hispanic population.^6,22,23^

Interestingly, ethnicity was not predictive of whether a patient with COVID-19 would be diagnosed inpatient versus the ED/outpatient setting. Given that ethnicity predicted testing delay and that testing delay predicted location of diagnosis, it is unclear why ethnicity would not also predict the location of diagnosis. There may be other unmeasured variables in the causal pathway not considered in our analysis. Other U.S.-based studies have shown ethnicity to predict hospitalization, with Hispanic individuals 2.8 times more likely to be hospitalized.^12^

Although non-Hispanic Black persons have been disproportionately impacted by COVID-19 in the United States, our analyses found a disproportionately low representation of non-Hispanic Black patients in the COVID-19 cohort.^1,2^ In addition, there was no significant difference in testing delay between non-Hispanic Black and non-Hispanic White patients. However, this secondary analysis involved a four-category race/ethnicity variable and a small sample size of non-Hispanic Black patients (*n*=67). Since Bexar County has low percentage of Black residents (8.6% vs. 12.8% nationally), and non-Hispanic Black patients had an even lower representation in our COVID-19 sample (*n*=163, 3.9%), other studies will be better equipped to explore impact of COVID-19 on non-Hispanic Black persons.^16,18^

Generalizability of results may be limited because data were collected from a single health system. Our data focus on the first 6 months of the pandemic and may not be generalizable beyond this time frame. Furthermore, some patients with COVID-19 evaluated during this time frame are not represented in the data due to missing ethnicity/race data in the EHR (*n*=433 of 4644, 9.32%). Despite these limitations, our findings clearly demonstrate a disproportionate burden of COVID-19 disease and delays in COVID-19 testing in Hispanic patients, even in a safety net system within a majority Hispanic community.

### Health equity implications

Because essential workers are more likely to be people of color already facing health care inequities, easing of COVID-19 protections is not race neutral and may further contribute to disparities. As such, policies should be in place to mitigate risk for minority communities through non-pharmaceutical interventions. These could include financial and practical support for individuals with COVID-19 in multigenerational households, job protections for doctor's visits or quarantine, and public health education interventions in minority communities.^24^ Delays in testing should be addressed by outreach to communities of color in historically underserved neighborhoods. In addition, policy attention should be focused on the persistent underlying inequities such as income, living situation, health insurance, structural racism, and health care access that perpetuate the health disparities found in this study. These efforts will support the development of more resilient, equitable communities and help preserve health during future public health crises.

## Abbreviations Used

CI: confidence interval
CIVOC: COVID-19 Infectious Disease Virtual Outpatient Clinic
ED: emergency department
EHR: electronic health record
OR: odds ratio
REDCap: Research Electronic Data Capture
SD: standard deviation
